# Scaling up health intervention: is planning in Nigeria becoming evidence based?

**DOI:** 10.4314/pamj.v10i0.72222

**Published:** 2011-10-03

**Authors:** Babayemi Oluwaseun Olakunde

**Affiliations:** 1 Wole-Ayo Hospital, P. O Box 1561, Ondo, Nigeria

**Keywords:** Evidence based planning, Nigeria, Cost analysis, Nigeria

## Abstract

With scaling up effective health interventions towards achieving the health-related Millennium Development Goals high on the policy agendas of many developing nations, the costs and as well as benefits of these health interventions are extremely vital in resource poor settings such as Nigeria. Despite the body of evidence on the significance of economic analysis, the integration of economic analysis into planning and decision making is limited in developing countries. This paper commented on the use of a cost analysis study for planning in Nigeria and need to institutionalize such practice.

## Background

With scaling up effective health interventions towards achieving the health-related Millennium Development Goals high on the policy agendas of many developing nations, the costs and as well as benefits of these health interventions are extremely vital in resource poor settings such as Nigeria [1]. The use of economic perspective as an approach for priority setting is of increasing acceptance as there is need for evidence based planning [2]. Economic analysis involves comparing the costs and benefits of different health interventions. While a cost analysis examines the economic inputs to an intervention, an economic evaluation examines both the cost and output of the same intervention [3,4].

Cost analysis is considered a sine qua non for health planning and budgeting. It is useful for estimating resources required to scaling up interventions, estimating the resources required to sustain intervention, advocacy, and resource mobilization [3,5]. Cost analysis is also used as a component for assessing health system performance [6].

Despite the body of evidence on the significance of economic analysis, planning and decision making is limited in developing countries [2,7]. Some of the many factors that have been attributed include lack of political willingness, shortage of technical expertise, and dearth of relevant data [2,7]. Perhaps, the situation in Nigeria is taking a paradigm shift.

## Planning of the ward minimum health care package

Primary health care (PHC) is the cornerstone of the Nigerian health system. However, in the last decades it has been in a dismal state, with a direct consequence on the overall performance of the health system [8]. With the aim of strengthening PHC, the National Primary Health Care Development Agency (NPHCDA) in 2007, after a detailed cost analysis, launched the Ward Minimum Health Care Package (WMHCP) for the plan period 2007-2012. The WMHCP consists of the following components: Control of Communicable Diseases (Malaria, Tuberculosis, STI/HIV/AIDS), Child Survival, Maternal and Newborn Care, Nutrition, Non-Communicable Diseases Prevention, and Health Education and Community Mobilization [9]. An initial costing exercise was conducted in one geopolitical zone, however, the stakeholders requested for further evaluation to cover all the six geopolitical zones. With technical support from Partnership for Transforming Health Systems (PATHS), a second survey in the five other geo-political zones was conducted and the cost for each of the components of WMHCP was evaluated. This was finally adopted by the stakeholders.

## Conclusion

This example shows the determination and commitment of the actors to integrate cost analysis into planning. It illustrates that policy is more likely to be evidence based if evidence is adequate and available at the time it is needed [10]. In addition to this, Nutbeam also stated that evidence that fits with political vision and balance of interests (or can be made to fit), evidence that points to actions for which the resources, capacity, system and infrastructure are more likely to inform policy [10].

However, evidence such as economic analysis alone doesn't make decisions. Other factors such as needs, values and equity have to be considered by decision makers, but the scientific basis to guide decision making should be the evidence [11]. The use of economic analysis to guide planning should be a continuous and an institutionalized exercise to reduce profligacy in countries with limited funds such as Nigeria. Further progress in Nigeria will require health researchers to recognise, understand, and engage more in health policy making process. Health researchers should undertake research relevant to local needs, establish the development of valid and reliable evidence (recognizing the value of systematic reviews and meta-analysis) [12].
